# Association between methylation quantitative trait loci and colorectal cancer risk, survival and cancer recurrence

**DOI:** 10.1038/s41416-025-03064-8

**Published:** 2025-06-12

**Authors:** Ines Mesa-Eguiagaray, Andrii Iakovliev, Xue Li, Maria Timofeeva, Yazhou He, Xiaomeng Zhang, Farhat V. N. Din, Susan M. Farrington, Athina Spiliopoulou, Malcolm G. Dunlop, Evropi Theodoratou

**Affiliations:** 1 Centre for Global Health, Usher Institute, University of Edinburgh, Edinburgh, UK; 2 Human Genetics Unit, Institute of Genetics and Cancer, University of Edinburgh, Edinburgh, UK; 3 School of Public Health and the Second Affiliated Hospital, Zhejiang University School of Medicine, Hangzhou, Zhejiang China; 4 Danish Institute for Advanced Study, Department of Public Health, University of Southern Denmark, Odense, Denmark; 5 Colon Cancer Genetics Group, Cancer Research UK Edinburgh Centre, Institute of Genetics and Cancer, University of Edinburgh, Edinburgh, UK; 6 Department of Oncology, West China School of Public Health and West China Fourth Hospital, Sichuan University, Chengdu, China; 7 Centre for Population Health Sciences, Usher Institute, University of Edinburgh, Edinburgh, UK

**Keywords:** Cancer epigenetics, Colorectal cancer

## Abstract

**Background:**

Epigenetic changes contribute to colorectal cancer (CRC) pathogenesis. We investigated whether methylation quantitative trait loci (mQTLs) are associated with CRC risk, survival and recurrence.

**Methods:**

Using a well-characterised Scottish case-control study (6821 CRC cases, 14,692 controls), we derived 118,982 mQTLs based on the Genetics of DNA Methylation Consortium (GoDMC). Association analysis between mQTLs and CRC risk, survival and recurrence was performed using logistic regression or Cox models respectively. Additionally, colocalisation analysis was performed.

**Results:**

19 mQTLs within 10 distinct genomic regions were associated with CRC risk. Two novel regions were mapped to *MDGA2* (*p* value = $3.0 \times\!{10}^{-6}$) and *STARD3* (*p* value = $5.6 \times\!{10}^{-6}$). Four regions mapped to *POU5F1B, POU2AF2 (c11orf53)/POU2AF3 (COLCA2), GREM1* and *CABLES2* were previously identified. Four regions mapped to *PPA2, PANDAR/LAP3P2, POU6F1* and *CTIF* contained SNPs previously identified by CRC GWAS but with SNPs annotated to different genes. We found no evidence that any of the 19 mQTLs associated with CRC risk influenced survival or recurrence after FDR correction. Colocalisation analysis suggested that in three of the ten regions the causal variants were shared for methylation and CRC risk.

**Conclusion:**

This study adds to the repertoire of CRC genes. However, we found no associations between methylation and CRC survival or recurrence.

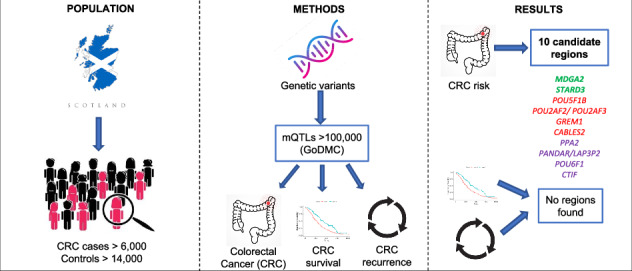

## Introduction

In the UK, over 40,000 cases of colorectal cancer (CRC) are diagnosed each year contributing to 11% of all cancers. Further, CRC is the second most common cause of cancer death [1]. The burden of CRC is expected to further increase with over 2 million cases globally by 2030 [1]. Many risk factors, including lifestyle and environmental factors, have been associated with CRC risk but there is also a contribution from genetic factors. The estimated heritability of CRC ranges from 16% (from common genetic variants identified in GWAS studies) to 35% (from twin studies) [2, 3].

The most recent meta-Genome-Wide Association Study (meta-GWAS) in CRC, which also included Transcriptome-Wide Association Study (TWAS) and Methylome-Wide Association Study (MWAS), identified 205 Single Nucleotide Polymorphisms (SNPs) mapped to 155 high-evidence effector genes (identified through functional analysis) associated with CRC risk [4]. However, the effect of those risk variants on CRC survival or recurrence has not been systematically investigated and fewer SNPs have been found to be associated with disease prognosis. To date, the GWAS catalog reports <100 variants associated with CRC prognosis and most of these associations were only observed for distal and proximal colon cancers [5, 6], reported in a single study [7, 8] and had *p*-values above the GWAS significant threshold. In a previous study using the Scottish Colorectal Cancer Study (SOCCS) data, genetic variants associated with CRC risk were not found to be associated with survival, either individually or combined in a polygenic risk score [9].

In this study, we aim to identify associations between quantitative trait loci for DNA methylation and CRC risk, and assess their effects on survival and recurrence. DNA methylation, a process that adds a methyl group to a cytosine phosphate guanine (CpG) site, plays an important role in the regulation of gene expression and in colorectal tumour carcinogenesis [10]. Further, disturbances in DNA methylation have been previously associated with all-cause mortality [11]. DNA methylation is influenced by aging, environmental exposures (smoking and alcohol consumption) but also by genetic factors. Methylation quantitative trait loci (mQTL) are genetic variants that influence DNA methylation levels across the genome. Previous post-GWAS approaches have mostly focused on expression quantitative trait loci (eQTL). However, recent studies suggest that mQTLs might shed new light on the epigenetic basis of several traits, and CRC in particular, thereby potentially identifying new candidate CRC risk genes [12].

Leveraging results from the largest meta-analysis of DNA methylation in blood to date from the Genetics of DNA Methylation Consortium (GoDMC) [13], complementary post-GWAS approaches using mQTLs could pinpoint further genetic loci for risk and especially for survival and recurrence. We hypothesise that mQTLs might be associated not only with risk but also with survival and recurrence in CRC patients which could shed light on disease aetiology and help identify targets for prevention, therapeutics and improved prognosis.

We used individual-level genome-wide data from case-control studies of CRC in Scotland and summary results from GoDMC to derive locus-specific mQTL genotypic scores and investigated their associations with CRC risk, survival and recurrence. In addition, we compared our findings with results from the most recent meta-GWAS of CRC risk and performed colocalisation analysis between mQTLs and CRC risk signals for the identified candidate regions, with the objective to discover if methylation changes and CRC risk share the same causal variants.

## Methods

### Study population

The study population was ascertained from a series of CRC population-based case-control studies recruited in Scotland, consisting of 6821 CRC cases and 14,692 controls [before quality control (QC) procedures]. Cases were obtained from the Colorectal Cancer Susceptibility Study (COGS) (*n* = 1012), Scottish Colorectal Cancer Study I and III [SOCCS (I and III)] (*n* = 4772 + 1037). Controls were obtained from Generation Scotland (GS) (*n* = 9937), from the Scottish Colorectal Cancer Study (SOCCS) (*n* = 2221), from the Lothian Birth Cohort (LBC) (*n* = 1522) and from the Colorectal Cancer Susceptibility Study (COGS) (*n* = 1012). Controls had no previous history of CRC. Information on age at diagnosis, sex and the American Joint Committee on Cancer (AJCC) stage was ascertained for each CRC case. After QC procedures (described below) and the exclusion of participants with missing covariates, 6379 CRC cases and 11,008 controls were included in the association analysis of DNA methylation scores and CRC risk. Further information on the studies used is provided in Supplementary Table 1.

Date and cause of death were ascertained from the Scottish Cancer Registry (SCR) following criteria that can be found elsewhere [9]. Patients were followed up until death or the end of the follow-up (July 1^st^ 2017-censored date). The final dataset of patients with available survival and genetic data contained 5921 cases. Recurrence was ascertained from the South East Scotland Database (SESCD) – a cancer database for the South East Scotland Region that covers Lothian, Fife, Dumfries and Galloway and the Borders NHS regions established in 2019. The SESCD is maintained by the Edinburgh Cancer Centre and contains data on demographics, diagnostic and clinical data, death records, follow-up data, treatments and comorbidities that dates back to 1974. More details (including a flowchart presented in supplementary Fig. 1) on the SESDC linkage procedures are given in supplementary methods. For patients with multiple diagnosis of CRC, only the first CRC diagnosis was retained as the primary cancer and recurrences were linked to that specific primary diagnosis. The final dataset with complete recurrence data and non-missing covariates contained 2045 CRC cases. Recurrence rates in SOCCS patients are presented in Supplementary Table 2.

### Genotyping, QC and imputation

Due to the longitudinal nature of these studies, CRC cases and controls samples were genotyped using different Illumina arrays: HumanHap240K or HumanHap300 for the COGS study; OmniExpressExome BeadChip (8v1.1, 8v1.2 or 8v1.3) or Omni5M for the SOCCS/GS study; and OmniExpressExome BeadChip (8v1.1, 8v1.2 or 8v1.3) or Human610-Quadv1 for the SOCCS/LBC study. Standard QC procedures were performed for each study. Individuals with a SNP call rate <95% or of non-European ancestry were excluded from the analysis. First-degree relative pairs were identified and for each pair we excluded the control (if there was any) or the individual with lower call rate (if they were both cases). SNPs with a minor allele frequency (MAF) < 0.05% were also excluded. Hardy-Weinberg equilibrium was assessed and SNPs that significantly deviated from it (*P* < 10^−5^) were also excluded. Further information on QC procedures can be found in Li et al. [14]. All QC analyses were performed using PLINK v1.9 [15].

Untyped SNPs were imputed using SHAPEITv2 and IMPUTEv2 with a merged reference panel using data from 1000 Genomes Project (phase 1, December 2013 release) [16] and UK10K (April 2014 release) [17]. SNPs with imputation quality information measure <0.80 were excluded. Imputed data from the three case-control studies were merged into a single dataset, retaining only variants present in all studies, and genetic principal components were calculated. SNPs on chromosome X or chromosome Y were not included in the analysis.

### Predicted DNA methylation

Genotypic scores reflecting the predicted level of methylation for a given probe were calculated for individuals in the CRC dataset using summary statistics for mQTLs from the GoDMC study and the GENOSCORES computational platform [18]. The GoDMC meta-analysis brought together data on 27,750 European ancestry individuals from 36 cohorts and have made summary statistics for *cis* and *trans* mQTLs publicly available. Cis-mQTLs refer to genetic variants that have an effect on DNA methylation locally, i.e. they are in close distance to the specific CpG site. In contrast, trans-mQTLs are located far from the affected site, even in a different chromosome. DNA methylation was measured using Illumina 450k or EPIC Beadchips in whole blood or cord blood.

Summary statistics for 182,196 methylation probes mapped to 31,259 unique genes were split into trait-associated regions of the genome. Trait-associated regions are defined as genomic regions that contain at least one SNP with $P \; < \;{1\times\!10}^{-6}$. A genotypic score was then computed for each region (locus), using all trait-associated SNPs in that locus with $P \; < \;{1\times\!10}^{-5}$. Each genomic region was separated by at least 1 Mb from another region. Genotypic scores were computed as a sum of the genotypes $(g)$ for an individual $(i)$, weighted by the effect size estimate $(\beta )$ for the trait of interest $(t)$ and further multiplied by the inverse of the SNP-SNP correlation matrix $\left(R\right)$, to adjust for linkage disequilibrium (LD). The formula to compute each score is:

$${score}\left(i,t\right)={g}_{i}{R}^{-1}{\beta }_{t}$$

The genotypic scores were then classified as cis (<50 kb), cis-x (between 50 kb and 5 Mb) or trans (>5 Mb) based on the distance from the transcription start and end sites of the corresponding genes. A total of 192,262 mQTLs were computed but scores that were highly correlated (>90% correlation) were excluded and 118,982 mQTLs (110,090 cis, 2905 cis-x and 5987 trans) mapped by distance to 25,756 unique genes were used in subsequent analyses.

The 1000 Genomes project panel restricted to European samples was used to adjust for LD [16]. For the computation of mQTL scores for chromosome 6, the human leucocyte antigen (HLA) region was excluded given the strong LD in this region.

### Statistical analysis

Logistic regression was used to estimate the effect of each mQTL score (one model per score) with CRC risk. The association between mQTL scores with overall survival (OS), CRC-specific survival (CSS) and recurrence free survival (RFS) was investigated using Cox proportional hazards models. For the CSS models, other cause of death was considered as a censored observation. RFS was considered as secondary outcome and defined as the time from primary treatment to date of recurrence. Recurrence was defined as any type of recurrence, which includes, local, regional and metastatic recurrence. For RFS models, patients with no recurrence were censored at date of death or at the end of follow-up. All models were adjusted for age, sex and ten principal components and stratified by AJCC stage (for survival and recurrence models). As supplementary CRC risk analysis, we investigated the association between mQTLs and CRC risk in patients with stage IV tumours at presentation vs the rest of stage tumours. These additional analyses aimed to identify genetic variants affecting DNA methylation that might play a role in late-stage tumour development and metastasis.

Multiplicity was corrected using the Benjamini–Hochberg method [19] for a False Discovery Rate (FDR) of 5%; and the regions of mQTL scores passing the significance threshold were selected as candidates for further analysis. FDR adjusted *p* values were calculated using *p.adjust* function in R. All analyses were conducted using R (version 4.1.0) in Eddie – a Linux environment developed by the University of Edinburgh.

### Colocalisation of mQTLs with CRC GWAS signals

In order to investigate whether mQTLs and CRC risk signals shared a common causal variant, colocalisation analysis was performed for the significant mQTLs within the identified candidate regions. The summary data was obtained from the newest meta-GWAS of CRC [4]. For each GWAS hit overlapping an mQTL, posterior probabilities of a shared causal variant were calculated using the Bayesian colocalisation test (based on the approximation of the Bayes factor) implemented in *coloc.abf()* function from the COLOC package in R. COLOC estimates posterior probabilities for 5 different hypotheses: Ho: no association, H1: mQTL association only, H2: CRC association only, H3: Both are associated but not colocalised, H4: Both are associated and colocalised. The boundaries of each candidate region were selected to contain all SNPs included in mQTL scores spanning that region. Colocalisation was based on the effect size estimates and standard errors and was restricted to a minimum of 10 variants in common between the methylation GWAS and the CRC risk GWAS. Regions with a probability of colocalisation (P4 > 0.80) were reported as regions with a shared causal variant between methylation and CRC risk. Supplementary colocalisation analysis looked at whether signals for any mQTL within an identified region (even if it was not statistically significant for its association with CRC risk) colocalised with signals from the CRC GWAS.

## Results

### Association with CRC risk

**Fig. 1:**
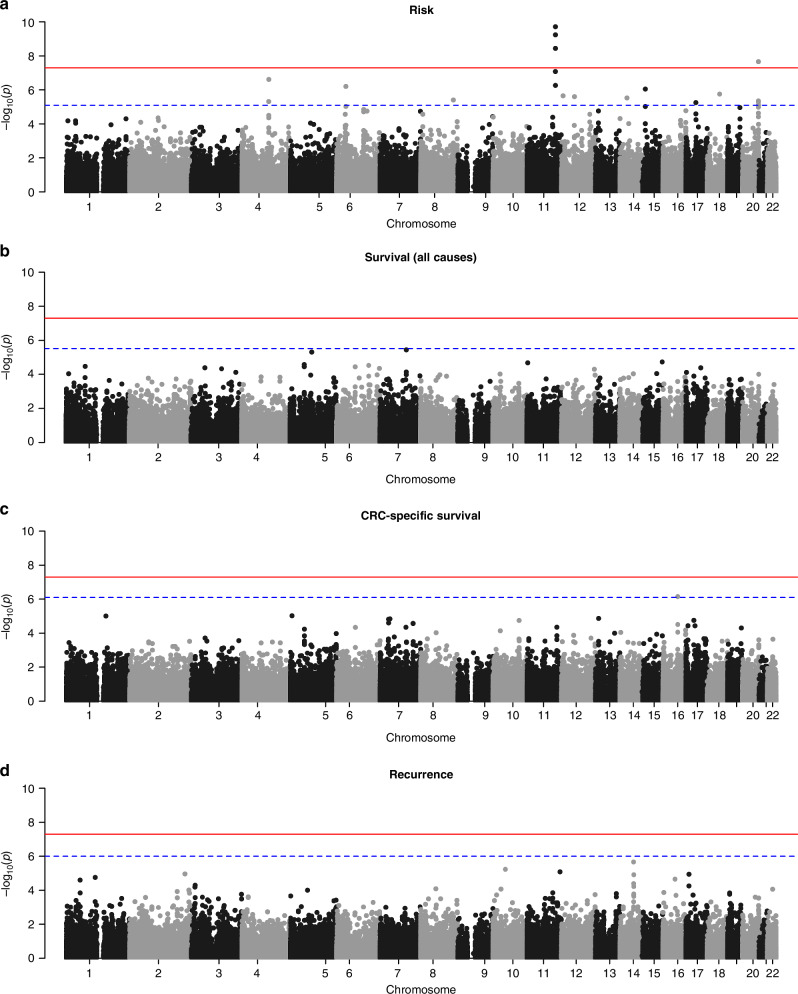
Manhattan plots for the association of mQTLs with CRC outcomes. Plots represent −log10 *P* values for mQTL associations with **A** risk, **B** all-cause survival, **C** CRC-specific survival and **D** recurrence. *P* values are ordered by chromosome and position (base pair). The horizontal red line denotes the threshold for genome wide significance (*p* < 5 × 10^-8^). The horizontal dashed blue line represents the FDR-adjusted significance threshold, which varies by outcome as it depends on the distribution and rank of the original *p* values.

A total of 6379 CRC cases [No.male (%) = 3633 (57%), mean age (SD) = 63[12]], 11,008 controls [No.male (%) = 5020 (46%), mean age (SD) = 59[12]] and 118,982 mQTL scores were included in the association analysis. A Manhattan plot of the *p* values for all associations between the mQTL scores and CRC risk is presented in Fig. 1a. A total of 19 mQTL scores were found to be associated with CRC risk after FDR correction (Table 1). Of the 19 statistically significant mQTLs, 18 were cis signals (cis-mQTLs). Five of the ten top cis-mQTLs scores were in chromosome 11 and tagged methylation levels of *POU2AF2* (previously known as *C11orf53*), *COLCA1* and *POU2AF3* (previously known as *COLCA2)* genes. Three cis-mQTLs were on chromosome 20 and tagged methylation of *LAMA5* and *CABLES2* genes. There were also two cis-mQTL scores tagging PPA2 gene (in chromosome 4) and two cis-mQTL tagging POU6F1 gene (in chromosome 12). We also found a single cis-mQTL in chromosomes 6 (PANDAR and LAP3P2 genes), 8 (PCAT1, POU5F1B and CASC8 genes), 15 (GREM1 gene), 17 (STARD3 gene) and 18 (CTIF gene). Additionally, we detected a statistically significant trans-mQTL score located in chromosome 20 (CABLES2 and LAMA5 genes region) that tagged methylation in STK10 gene (chromosome 5) that was associated with CRC risk. Those 19 mQTLs were located in 10 distinct candidate regions of the genome and were taken forward for subsequent analyses. Supplementary risk analysis restricting the cohort to CRC patients with stage IV at presentation did not find any significant associations with methylation signals (Supplementary Table 3) suggesting that there are no specific signals associated with advanced disease or the development of metastasis.

**Table 1:** Significant associations between mQTL scores and CRC risk.

Chromosome	Region^a^	Gene(s) tagged by probe	QTL type	Methylation probe	Number of SNPs in mQTL score	Estimate	Standard error	*P* value	*P* value, FDR corrected
4	104,940,737- 105,558,268	PPA2	cis cis	cg21545890	22	−1.18	0.23	2.44 × 10^-7^	4.84 × 10^-3^
				cg09289469	333	0.39	0.09	4.83 × 10^-6^	3.48 × 10^-2^
6	36,402,477-36,758,526	PANDAR and LAP3P2	cis	cg02730678	262	−0.27	0.05	6.29 × 10^-7^	9.35 × 10^-3^
8	127,383,349- 127,454,743	POU5F1B, CASC8 and PCAT1	cis	cg14289643	100	−0.41	0.09	3.93 × 10^-6^	3.34 × 10^-2^
11	111,120,465- 111,805,145	*POU2AF2 (*C11orf53)	cis cis	cg25129781	112	0.94	0.15	1.92 × 10^-10^	2.28 × 10^-5^
				cg08142096	1	−1.71	0.29	3.59 × 10^-9^	1.42 × 10^-4^
		COLCA1 and *POU2AF3 (*COLCA2)	cis cis cis	cg09213929	65	0.71	0.12	5.74 × 10^-10^	3.41 × 10^-5^
				cg10045354	190	−0.30	0.06	8.18 × 10^-8^	1.95 × 10^-3^
				cg21645554	196	−0.41	0.08	5.46 × 10^-7^	9.28 × 10^-3^
12	50,665,404- 51,372,735	Unknown	cis	cg25366390	63	0.27	0.06	2.23 × 10^-6^	2.41 × 10^-2^
		POU6F1	cis	cg22163059	168	0.51	0.11	2.50 × 10^-6^	2.48 × 10^-2^
14	47,180,232- 47,709,403	MDGA2	cis	cg22964621	596	0.49	0.10	2.97 × 10^-6^	2.72 × 10^-2^
15	32,694,234- 32,814,035	GREM1	cis	cg21924449	103	0.84	0.17	8.96 × 10^-7^	1.18 × 10^-2^
17	38,742,575- 40,487,962	STARD3	cis	cg00129232	1595	0.13	0.03	5.58 × 10^-6^	3.69 × 10^-2^
18	48,839,047- 48,934,015	CTIF	cis	cg13840032	10	−0.92	0.19	1.77 × 10^-6^	2.11 × 10^-2^
20	61,913,515- 62,472,409	CABLES2	cis cis	cg22601191	499	−0.31	0.06	2.15 × 10^-8^	6.40 × 10^-4^
				cg01379171	17	−0.91	0.20	4.97 × 10^-6^	3.48 × 10^-2^
		LAMA5	cis	cg22307297	485	−0.20	0.04	4.47 × 10^-6^	3.48 × 10^-2^
		STK10 (CHR5)	trans	cg26561207	26	−0.73	0.16	7.52 × 10^-6^	4.71 × 10^-2^

^a^Largest region that includes all SNPs within those mQTL scores. Models presented were fitted in 6379 CRC cases and 11,008 controls and adjusted for age, sex and 10 principal components.

*FDR* false discovery rate, *QTL* quantitative trait loci, *SNP* single nucleotide polymorphism.

### Association with CRC survival and recurrence

A total of 5921 CRC cases had follow-up data for the survival analyses. Of these, 1994 (34%) CRC cases died during follow-up, with 1415 (71% of those who died) having CRC as the primary cause of death. A total of 2045 CRC cases had linkage data for the recurrence analysis, with recurrence occurring in 942 (46%) of these patients. Results for the association of the 19 mQTLs identified for risk in the 10 candidate regions with survival or recurrence are presented in Table 2. None of the 19 mQTLs associated with risk were associated with CRC survival (all-cause or CRC-specific) or recurrence after FDR correction. The mQTL tagged to STARD3 gene suggested an association with CRC- specific survival [HR = 1.14 (1.05, 1.24), *P* value = 0.007] that was no longer statistically significant after correcting for multiple testing.

**Table 2:** Results for the association between mQTLs and CRC survival (OS and CSS) and recurrence (RFS) for the 10 candidate regions identified in which 19 mQTLs were associated with CRC risk.

Chr	Region^a^	Gene(s)	Methylation probe	OS deaths/total		CSS CRC deaths/total		RFS Recurrent events/total	
				**1994/5921**		**1415/5921**		**942/2045**	
				**HR (95% CI)**	***P*** **value**	**HR (95% CI)**	***P*** **value**	**HR (95% CI)**	***P*** **value**
4	104,940,737- 105,558,268	PPA2	cg21545890	1.22 (0.58, 1.85)	0.543	0.82 (0.06, 1.58)	0.610	1.28 (0.36, 2.19)	0.601
			cg09289469	1.07 (0.83, 1.30)	0.590	1.24 (0.97, 1.52)	0.122	0.87 (0.53, 1.20)	0.410
6	36,402,477-36,758,526	PANDAR and LAP3P2	cg02730678	1.08 (0.93, 1.23)	0.327	1.06 (0.89,1.24)	0.483	1.08 (0.87, 1.30)	0.462
8	127,383,349- 127,454,743	POU5F1B, CASC8 and PCAT1	cg14289643	1.04 (0.80, 1.28)	0.746	1.06 (0.77, 1.34)	0.700	1.04 (0.69, 1.39)	0.822
11	111,120,465- 111,805,145	*POU2AF2 (*C11orf53)	cg25129781	0.85 (0.45, 1.24)	0.414	0.87 (0.41,1.34)	0.575	0.76 (0.19, 1,32)	0.338
			cg08142096	1.35 (0.56, 2.13)	0.454	1.08 (0.15, 2.01)	0.877	1.19 (0.06, 2.31)	0.767
		COLCA1 and *POU2AF3 (*COLCA2)	cg09213929	0.81 (0.49, 1.12)	0.183	0.84 (0.47, 1.21)	0.355	0.74 (0.28, 1.19)	0.186
			cg10045354	1.06 (0.92, 1.21)	0.412	1.03 (0.85, 1.20)	0.748	1.07 (0.86, 1.28)	0.508
			cg21645554	1.06 (0.88, 1.28)	0.574	1.01 (0.75, 1.27)	0.955	1.09 (0.78, 1.40)	0.577
12	50,665,404- 51,372,735	POU6F1	cg25366390	1.38 (0.35, 2.42)	0.538	1.40 (0.19, 2.62)	0.583	1.77 (0.26, 3.28)	0.461
			cg22163059	1.22 (0.94, 1.50)	0.156	1.13 (0.80, 1.47)	0.463	0.93 (0.53, 1.34)	0.733
14	47,180,232- 47,709,403	MDGA2	cg22964621	1.10 (0.81, 1.39)	0.513	1.07 (0.73, 1.41)	0.698	1.27 (0.85, 1.68)	0.267
15	32,694,234- 32,814,035	GREM1 and AC090877.2	cg21924449	1.36 (0.91, 1.81)	0.180	1.33 (0.80, 1.87)	0.292	1.20 (0.55, 1.84)	0.586
17	38,742,575- 40,487,962	STARD3	cg00129232	1.06 (0.98,1.14)	0.168	1.14 (1.05, 1.24)	0.007^b^	0.89 (0.78, 1.01)	0.060
18	48,839,047- 48,934,015	CTIF	cg13840032	1.12 (0.60, 1.64)	0.674	1.15 (0.53, 1.77)	0.662	1.26 (0.49, 2.02)	0.561
20	61,913,515- 62,472,409	CABLES2	cg22601191	0.87 (0.72, 1.03)	0.084	0.89 (0.71, 1.08)	0.230	0.94 (0.72,1.16)	0.577
			cg01379171	0.63 (0.08, 1.18)	0.104	0.64 (0.01, 1.29)	0.179	0.73 (0.00, 1.53)	0.443
		LAMA5	cg22307297	0.97 (0.84, 1.09)	0.573	0.92 (0.77, 1.07)	0.255	1.00 (0.83, 1.17)	0.982
			cg26561207	0.86 (0.40, 1.31)	0.503	0.84 (0.29, 1.38)	0.518	0.84 (0.19, 1.49)	0.599

*Chr* chromosome, *CI* Confidence Interval, *CRC* colorectal cancer, *CSS* Colorectal cancer specific survival, *HR* Hazard Ratio estimated using Cox proportional models adjusted for age, sex and 10 PCs and stratified by AJCC stage, *OS* Overall Survival, *RFS* Recurrence free survival.

^a^Largest region that includes all SNPs within those mQTL scores. Total number of models fitted per outcome is 19 (one model for each of the 19 mQTL scores found to be associated with CRC risk).

^b^P value not significant after FDR and Bonferroni correction, threshold for statistical significance with 19 tests would be *p* < 0.0026 after Bonferroni correction.

Analyses looking at the association of mQTLs in all regions of the genome with survival [all-cause of death (Fig. 1b) and CRC-specific death (Fig. 1c)] and recurrence (Fig. 1d) did not show any significant results after FDR correction (Supplementary Tables 4–6). A *cis*-mQTL score in chromosome 16 tagging methylation of the *KATNB1* gene had a suggestive association to CRC-specific survival (*P* value = 7.11E−07, FDR corrected *P* value of 0.08).

### Colocalisation of mQTL identified candidate regions with CRC meta-GWAS signals

Colocalisation analyses results for the 19 mQTLs found to be associated with CRC risk within the 10 identified candidate regions are shown in Table 3. Of the ten regions identified for the risk analysis, three regions were found to colocalise with signals from meta-GWAS in CRC. Those were the regions in chromosomes 6 (mapped to *PANDAR/LAP3P2*), 11 (mapped to *POU2AF2*, *COLCA1* and *POU2AF3*), and 20 (mapped to *CABLES2* and *LAMA5*). Results for the PPA2 region (PPH4 = 0.51) were suggestive of colocalisation but did not meet the predefined threshold (PPH4 > 0.80). The regions in chromosomes 8, 12, 15, 17 and 18 had a high PP of H3 suggesting an association with both mQTLs and CRC meta-GWAS signals but no shared causal variants. Finally, the region in chromosome 14 mapped to *MDGA2* gene suggested only an association with methylation signals. Additional colocalization analyses for all mQTL scores within the candidate regions (Supplementary Tables 7–15) found three more regions for which mQTLs and meta-GWAS results shared a causal variant. Those were regions in chromosome 12 (mapped to *POU6F1*), 15 (mapped to *GREM1*) and 17 (mapped to *STARD3*).

**Table 3:** Colocalisation of the 19 mQTLs and CRC meta-GWAS data for the ten candidate regions identified.

Chr	Region^a^	Gene(s)	Methylation probe	No. SNPs in common with meta-GWAS	PP H0	PP H1	PP H2	PP H3	PP H4	Top colocalised SNP(s)
4	104,940,737- 105,558,268	PPA2	cg21545890	22	<0.01	0.39	<0.01	0.10	**0.51**	
			cg09289469	318	<0.01	<0.01	<0.01	**1.00**	<0.01	
6	36,402,477-36,758,526	PANDAR and LAP3P2	cg02730678	274	<0.01	<0.01	<0.01	0.06	**0.94**	rs9470361
8	127,383,349- 127,454,743	POU5F1B, CASC8 and PCAT1	cg14289643	92	<0.01	<0.01	<0.01	**1.00**	<0.01	
11	111,120,465- 111,805,145	*POU2AF2 (*C11orf53)	cg25129781	111	<0.01	<0.01	<0.01	0.18	**0.82**	rs3087967
			cg08142096	<5	Not enough common SNPs found					
		COLCA1 and *POU2AF3 (*COLCA2)	cg09213929	74	<0.01	<0.01	<0.01	0.32	**0.68**	rs4608113
			cg10045354	186	<0.01	<0.01	<0.01	0.08	**0.92**	rs7130173
			cg21645554	193	<0.01	<0.01	<0.01	<0.01	**0.99**	rs3087967
12	50,665,404- 51,372,735	POU6F1	cg25366390	<5	Not enough common SNPs found					
			cg22163059	163	<0.01	<0.01	<0.01	**1.00**	<0.01	
14	47,180,232- 47,709,403	MDGA2	cg22964621	586	<0.01	**0.99**	<0.01	<0.01	<0.01	
15	32,694,234- 32,814,035	GREM1 and AC090877.2	cg21924449	102	<0.01	<0.01	<0.01	**1.00**	<0.01	
17	38,742,575- 40,487,962	STARD3	cg00129232	1606	<0.01	<0.01	<0.01	**0.99**	<0.01	
18	48,839,047- 48,934,015	CTIF	cg13840032	10	<0.01	<0.01	<0.01	**1.00**	<0.01	
20	61,913,515- 62,472,409	CABLES2	cg22601191	537	<0.01	<0.01	<0.01	0.05	**0.94**	rs1741640
			cg01379171	17	<0.01	<0.01	<0.01	0.02	**0.98**	rs2427313
		LAMA5	cg22307297	471	<0.01	<0.01	<0.01	0.06	**0.94**	rs1741640
			cg26561207	<5	Not enough common SNPs found					

PP = posterior probability for each one of the hypothesis tested calculated with colco.abf() function. Probabilities in bold  represent the highest PP hypothesis for that probe.

^a^Largest region that includes all SNPs within those mQTL scores.

**Fig. 2:**
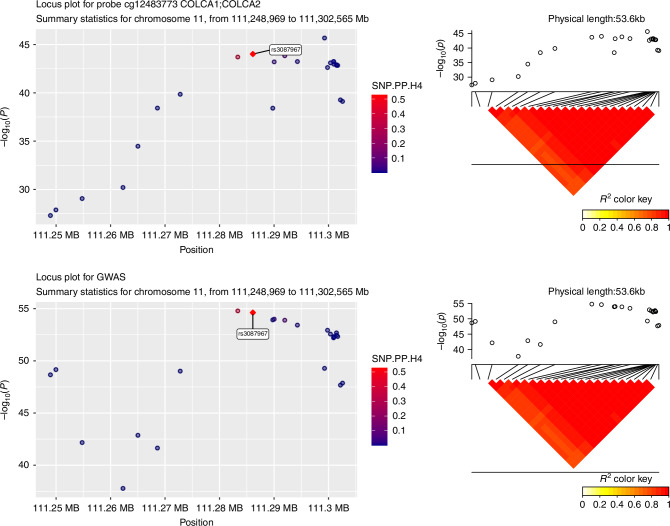
Locus plots for probe cg12483773 and meta-GWAS data within POU2AF2 (C11orf53), COLCA1 and POU2AF3 (COLCA2) genes that were found to colocalised. A diagram of the LD pattern is also presented. Top colocalised SNP is rs3087967.

An example of the locus plot for one of the colocalised regions (mQTL for probe cg12483773 in *POU2AF2*, COLCA1 and *POU2AF3* region) with the CRC meta-GWAS data is presented in Fig. 2 where we observe that mQTL for probe cg12483773 and CRC risk signals shared a common causal variant (rs3087967). Locus plots for the rest of the regions are presented in Supplementary Figs. 2–6.

## Discussion

This study identified ten genomic regions harbouring mQTLs that are associated with CRC risk. Comparing our results with the most recent CRC meta-GWAS study (Supplementary Table 16) that integrated results from TWAS and MWAS, we found two new regions (mapped to genes *MDGA2* and *STARD3*) that have not previously been identified. For these regions there were no individual SNPs or effector genes listed in the meta-GWAS. However, a SNP (rs2313171) in the *STARD3/PGAP3/ERBB2* genes region was identified in TWAS and a CpG site in *PGAP3* was identified in MWAS but after conditional analyses on the GWAS associations within 1 Mb, these were no longer considered as novel risk associations.

*MDGA2* a cell adhesion molecule that belongs to the brain immunoglobulin superfamily, is involved in inhibitory synapse regulation and in axonal growth [20]. *MDGA2* is a tumour suppressor for gastric cancer and hypermethylation of this gene has been associated with gastric cancer prognosis [21]. More recently, *MDGA2* has also been postulated as a relevant diagnostic and prognostic biomarker for breast [22] and nasopharyngeal cancers [23].

*STARD3* is a member of the lipid transfer proteins (LTP) STAR family involved in the regulation of the accumulation of cholesterol in endosomes and in mediating its distribution [24]. Several studies have found an association of *STARD3* with different cancer types, including lung [25], pancreatic [26], gastric [27] ovarian [28] and especially breast cancers for which high *STARD3* expression is associated with recurrence, metastasis and poor prognosis [29, 30]. Further, *STARD3* is considered a potential oncogene with an inhibitor already reported [31].

Four of the candidate regions that we identified (mapped to *POU5F1B, POU2AF2/ POU2AF3, GREM1* and *CABLES2*), were also identified previously [4].

*POU5F1B* is a retrogene which only appears in descendants of great apes, hence its study with animal models is very limited. Despite this, previous studies have linked this gene to breast, colon and prostate cancers [32, 33] and a recent study found that DNA methylation levels at this gene in human blood are associated with cancer proliferation and negative prognosis in CRC patients [32, 34].

*POU2AF2* (previously known as *C11orf53*) is a protein-coding gene that acts as coactivator of the POU2F3 that regulates tuft cells in small cell lung cancer and colorectal cancer [35, 36]. *POU2AF2* and *COLCA1/POU2AF3* are located within the same region (11q23.1). *POU2AF3* and *COLCA1* are co-regulated genes. *POU2AF3* is expressed in epithelial, immune and other cells, as well as in tumour cells. *POU2AF3* expression is reduced in tumour cells which might supress its formation providing protection [37].

*GREM1* encodes a protein of the bone morphogenic antagonistic family and has been linked to inflammation [38], fibrosis of numerous organs and cancers, including CRC [39]. Overexpression of *GREM1* in CRC patients is associated with tumour proliferation [39], polyposis [40] and poor prognosis [41].

*CABLES2* is a protein-coding gene that interacts with other CABLES family members [42]. Recent studies investigating gene expression have found that *CABLES2* is a tumour suppressor involved in the apoptosis pathway and plays an important role in CRC carcinogenesis [43]. In the *CABLES2/LAMA5* region we also found a trans-mQTL for *STK10* gene (on chromosome 5) that might be suggestive of a relationship between the SNPs affecting methylation levels in both *CABLES2*/*LAMA5* and *STK10* gene. *STK10* gene is involved in inhibiting cellular death in cancerous cells and has been found to be highly expressed in multiple tissues, including the colon [44]. Further, a recent study found five genetic loci (one of them in *STK10* gene) that interact with fine particulate matter (PM_2.5_) concentration and are associated with CRC risk and DNA methylation might be the driving mechanism [45].

The remaining four regions identified in our analysis overlapped with SNPs found in meta-GWAS within that same region but a different effector gene was identified. SNPs within our candidate regions in chromosome 4 (mapped to *PPA2* gene), chromosome 6 (mapped to *PANDAR/LAP3P2*) chromosome 12 (mapped to *POU6F1*) and chromosome 18 (mapped to *CTIF* gene), were assigned to genes TET2, CDKN1A, LIMA1 and ACAA2 respectively and as effector genes in the CRC risk meta-GWAS.

Given the limited information on the genetic susceptibility to CRC survival and recurrence, this study also investigated the association of mQTLs with those outcomes. The results showed that, after FDR correction, associations between the 19 mQTLs found and survival (all-cause and CRC-specific) and recurrence were not statistically significant. However, the mQTL tagged to the STARD3 gene suggested an association with CRC- specific survival that was no longer significant after correcting for multiple testing. Additionally, we found one mQTL score in chromosome 16 that mapped to the *KATNB1* gene that we believe might be of interest as it was suggestive of an association with CRC-specific survival (*p* value = 0.08 after FDR). *KATNB1* encodes the Katanin p80 protein that is a member of the AAA family of ATPases involved in microtubule disassembly at centrosomes [46]. Katanin p80 has been found to correlate with lymph node metastasis and worse survival in breast cancer and non-small-cell lung cancer patients [47] and with shorter disease-free survival in papillary thyroid cancer patients [48].

In order to investigate whether the identified mQTLs and signals from CRC meta-GWAS overlap, we performed colocalisation analysis using summary statistics from GoDMC and CRC risk meta-GWAS. Of the ten regions identified for risk, we found evidence that mQTLs in three regions (*PANDAR/LAP3P2* in chromosome 6, *POU2AF2 /COLCA1/ POU2AF3* in chromosome 11 and *CABLES2/LAMA5* in chromosome 20) colocalise with GWAS signals suggesting that DNA methylation might be involved in CRC susceptibility. Additional analyses (for probes not statistically significant for CRC risk) identified three more regions (*POU6F1* in chromosome 12, *GREM1* in chromosome 15 and *STARD3* in chromosome 17) with evidence of a shared variant influencing both DNA methylation and CRC risk. We note that colocalisation analysis alone cannot establish that the effect of these shared genetic variants on CRC is through methylation, but highlights this as a possible mechanism.

Our study has important strengths, we integrate case-control studies of CRC patients in Scotland from a defined population with detailed phenotyping and long term follow up. We also leverage the use of the GENOSCORES platform to derive mQTLs using the GoDMC reference panel with over 30,000 participants and over 400,000 DNA methylation sites in blood (including ~9% long-range [trans] associations) and that it is the largest study to date on DNA methylation in blood [13]. The approach of using DNA sequence proxies for methylation holds considerable promise as a hypothesis-generating tool, and may also help in identifying diagnostic and prognostic biomarkers.

This study also has some limitations. Our dataset did not have complete information of possible confounders, such as smoking or alcohol consumption, which could be driving some of the associations observed between mQTLs and CRC risk, so we were unable to explore the environmental factors or adjust for them. In our study, although the risk analysis had sufficient power to detect an association between mQTLs and CRC risk, the analysis for survival and recurrence was underpowered (particularly when looking at all regions of the genome). Further, survival and recurrence data are more prone to bias (particularly selection and lead-time bias). In our study, selection bias may be present as patients included in the recurrence dataset were more likely to have been seen by an oncologist, had higher-stage tumours and higher recurrence rates, hence they might not be representative of all CRC cases. Moreover, lead-time bias might be present if earlier detection of CRC led to improved survival. These biases, along with the mentioned limitations in statistical power, may have masked some of the true associations between mQTLs and CRC survival and recurrence.

Furthermore, our methylation proxies were measured in blood and not in colon tissue. Previous studies for breast cancer, have shown that methylation measured in surrogate sample tissues (such as cervical tissue as surrogate for breast tissue) might be better proxies to estimate cancer risk than methylation measured in blood [49]. However, recent studies demonstrate that blood might be a good proxy for colon with mQTL correlation between both tissues estimated to be over 60% [12]. Future research should aim to address these limitations, with studies based on large sample sizes with detailed follow-up and environmental exposure data and methylation measured in colon tissue.

In summary, the results from this study, although in need of replication, could be a first step to investigate the role of these genes (and their methylation levels) in CRC cancer susceptibility and prognosis and as potential biomarkers for prevention, diagnosis and treatment. Of special importance is the discovery of two genes *MDGA2* and *STARD3* for which significant associations with many other tumour types have been previously found. These genes warrant further investigation to inform their potential as biomarkers, particularly considering the previous success in targeting and inhibiting *STARD3* in other cancers.

## Supplementary information

Supplemental Materials for the main article

## Data Availability

The individual-level datasets used and/or analysed during the current study are available from the corresponding author on reasonable request. The mQTLs were derived from the GoDMC consortium (http://mqtldb.godmc.org.uk/) and CRC summary statistics from meta-GWAS by Fernandez-Rozadilla et al. (10.1038/s41588-022-01222-9).
