# Supplementary material for: Association between methylation quantitative trait loci and colorectal cancer risk, survival and cancer recurrence
Source: Br J Cancer. 2025 Jun 12;133(4):564–73. doi: 10.1038/s41416-025-03064-8 (PMC12356881; doi:10.1038/s41416-025-03064-8)
Supplement: Supplementary file 1 — Supplemental Materials for the main article [file 41416_2025_3064_MOESM1_ESM.docx]

**TITLE:** Association between methylation quantitative trait loci and colorectal cancer risk, survival and recurrence in case-control studies of colorectal cancer in Scotland.

**Authors:**

Ines Mesa Eguiagaray, Andrii Iakovliev, Xue Li, Maria Timofeeva, Yazhou He, Xiaomeng Zhang, Farhat V. N. Din, Susan M. Farrington, Athina Spiliopoulou, Malcolm G. Dunlop, Evropi Theodoratou

# Supplementary Methods

## Linkage of SOCCS studies to SESCD

An initial cohort (n=14,048) was ascertained from SESCD containing all CRC cases [defined as C18 to C20 in the International Classification of Diseases version 10 (ICD10)] present at the time of extraction (June 2021). Linkage of the SESCD cohort with the cases in the Scottish studies was based on the Community Health Index (CHI) number which uniquely identifies all Scottish residents registered with a general practitioner (GP) in Scotland. CHI number was available for 5,534 CRC cases in SOCCS. A total of 3,066 CRC diagnoses (in 2,926 patients) were matched after linkage. A considerable number of patients (n=1,605) were diagnosed or living outside of the South East Scotland regions, hence could not be matched. The rest of the patients unmatched are assumed to not have been seen by an oncologist and therefore they are not included in the SESCD.

Some patients (n=133) had multiple CRC diagnoses recorded, hence in order to link the same tumour diagnosis from both databases we checked date of diagnosis and data on tumour characteristics and those cases with dates within 6 months with the same clinical data were considered the same diagnosis. For those cases with missing date of diagnosis further linkage to the Scottish Cancer Registry (SMR06) helped to identify whether the diagnosis was the same in both SESCD and SOCCS databases.

A flowchart for the linkage of the SESCD and SOCCS databases is presented in **supplementary Figure 1.**

## Recurrence definition

Recurrence was defined as any type of recurrence which includes local, regional and metastatic/distant recurrence. Local recurrence is when the cancer is in the same place as the original cancer or very close to it. Regional recurrence occurs if the cancer has spread to the lymph nodes or tissues near the original cancer. Metastatic or distant metastasis is when the cancer has spread to other organs or tissues that are far from the original cancer. Recurrence status and recurrence date were derived from multiple variables within the SESCD, hence some recurrence dates for the same tumour differed (n=131, 6%) and in that case, the first recurrence date was taken. For many tumours, particularly those with local or regional recurrence, date of recurrence was missing (n=1,268, 62%) and the date of follow-up was taken as recurrence date.

Supplementary Figure 1 Flowchart for the linkage of SOCCS and SESCD and creation of the final recurrence dataset used in the study

## Linkage

**South East Scotland Database (SESCD)**

n= 14,048 CRC cases

**Scottish Cancer of Colorectal Studies (SOCCS)**

n= 6,821 CRC cases

**Linkage using CHI numbers**

n= 3,066 CRC diagnoses

n=2,926 CRC cases

**Unmatched reasons**

- Outside of south East Scotland (n=1,605)
- Unavailable CHI (n=395)
- Unknown (n=1,895)

**Additional linkage to SMR06**

n= 3,173 CRC diagnoses

n=2,979 CRC cases

**Exclusion criteria:**

- Not the same diagnosis (n=49)
- Not follow-up data on survival (n=816)
- Missing covariate data (n=69)

**Final Recurrence Dataset**

n=2,045 CRC cases

# Supplementary Tables

## Supplementary Table 1: Studies included in the population samples with genotyping array information

| **Study** | **Setting** | **Participants** | **Sampling** | **Genotyping array** | **Study Cohort*** |
| --- | --- | --- | --- | --- | --- |
| Scotland1 | COGS (Colorectal Cancer Susceptibility Study) | 1,012 cases; 1,012 controls | Population-based incidence cases aged < 55 years at diagnosis in Scotland; population-based controls frequency matched by area of residence within Scotland. | Illumina HumanHap 240K and 300 | 6,379 cases; 11,008 controls |
| SOCCS/GS | Scottish Colorectal Cancer Study 1 (SOCCS), Generation Scotland (GS) | 4,772 cases; 12,158 controls (9,937 from GS and 2,221 from SOCCS) | Population based incident cases (SOCCS 1), and population-based controls from Scotland (GS and SOCCS 1 controls). | OmniExpressExome BeadChip 8v1.1 or 8v1.2 or 8v1.3, Omni5M |  |
| SOCCS/LBC | Scottish Colorectal Cancer Study 3 (SOCCS3), Lothian Birth Cohort | 1,037 cases; 1,522 controls | Population based incidence cases from Scotland and population-based controls from the Lothian Birth Cohorts 1921 and 1936. | OmniExpressExome BeadChip 8v1.1 or 8v1.2 or 8v1.3, Illumina610-Quadv1 |  |

*Number of samples after QC procedures and removing samples with missing phenotype data. Updated from Li et al. (1)

## Supplementary Table 2 Recurrence rates in the series of case-control studies in Scotland (N=2,662)

| Type of recurrence | Freq | % |
| --- | --- | --- |
| Local |  |  |
| Yes | 603 | 23 |
| No | 2,059 | 77 |
| Regional |  |  |
| Yes | 392 | 15 |
| No | 2,270 | 85 |
| Local and/or Regional |  |  |
| Yes | 712 | 27 |
| No | 1,950 | 73 |
| Distant |  |  |
| Yes | 895 | 34 |
| No | 1,767 | 66 |

## Supplementary Table 3 Associations between mQTLs and CRC risk in participants diagnosed with a stage IV CRC at presentation.

| **Chromosome** | **Region** | **Gene** | **mQTL type** | **Coefficient** | **SE** | **P value** | **P, FDR** |
| --- | --- | --- | --- | --- | --- | --- | --- |
| 13 | 112677895 - 113095819 | MCF2L | cis | 0.29 | 0.06 | 3.99E-06 | 0.48 |
| 10 | 101797338 - 103101176 | ELOVL3 | cis | -1.26 | 0.29 | 1.28E-05 | 0.63 |
| 12 | 123998986 - 124154046 | DNAH10 | cis | -3.03 | 0.71 | 2.02E-05 | 0.63 |
| 4 | 133861248 - 134454199 | PABPC4L | cis | 1.92 | 0.46 | 2.88E-05 | 0.63 |
| 13 | 112924107 - 112952958 | MCF2L | cis | 2.18 | 0.52 | 3.09E-05 | 0.63 |
| 1 | 203399089 - 203768908 | Unknown | - | 0.38 | 0.09 | 3.28E-05 | 0.63 |
| 10 | 133182657 - 133243436 | Unknown | - | 1.40 | 0.34 | 3.73E-05 | 0.63 |
| 16 | 84340348 - 84403003 | ATP2C2 | cis | 0.82 | 0.20 | 4.51E-05 | 0.67 |
| 18 | 62495558 - 62599770 | TASP1 | trans | -1.35 | 0.33 | 5.55E-05 | 0.73 |
| 3 | 41030640 - 41084235 | Unknown | - | -1.23 | 0.31 | 8.86E-05 | 0.99 |

Associations are ordered by P value (from lowest to highest) and only those with $P<{1 x 10}^{-4}$ before FDR correction are presented. FDR=False Discovery Rate, HR=Hazard Ratio, SE=standard error.

## Supplementary Table 4 Associations between mQTLs and all-cause survival for participants diagnosed with CRC.

| **Chromosome** | **Region** | **Gene** | **mQTL type** | **Coefficient** | **HR** | **SE** | **P value** | **P, FDR** |
| --- | --- | --- | --- | --- | --- | --- | --- | --- |
| 7 | 102800397 - 103418273 | DNAJC2, PSMC2 | cis | -1.80 | 0.17 | 0.39 | 3.59E-06 | 0.29 |
| 5 | 84294309 - 84636751 | EDIL3, EDIL3-DT | cis | -1.26 | 0.29 | 0.27 | 4.86E-06 | 0.29 |
| 5 | 56188249 - 56266635 | Unknown | - | -3.93 | 0.02 | 0.88 | 8.55E-06 | 0.33 |
| 16 | 86201922 - 86307154 | Unknown | - | 0.41 | 1.51 | 0.10 | 1.51E-05 | 0.33 |
| 15 | 98921754 - 99057801 | IGF1R, SYNM-AS1 | cis | -0.62 | 0.54 | 0.14 | 1.89E-05 | 0.33 |
| 11 | 3136379 - 3146592 | OSBPL5 | cis | 1.46 | 4.31 | 0.34 | 2.13E-05 | 0.33 |
| 5 | 54967862 - 56081174 | MCIDAS | cis | 0.28 | 1.32 | 0.07 | 2.69E-05 | 0.33 |
| 6 | 126356313 - 127906954 | ECHDC1 | cis | 0.16 | 1.17 | 0.04 | 3.01E-05 | 0.33 |
| 1 | 76091265 - 76209483 | ST6GALNAC5 | cis | 1.74 | 5.69 | 0.42 | 3.44E-05 | 0.33 |
| 5 | 55038815 - 55807173 | CDC20B, GPX8 | cis | 0.76 | 2.14 | 0.18 | 3.58E-05 | 0.33 |
| 1 | 228881168 - 229203560 | Unknown | - | -1.03 | 0.36 | 0.25 | 3.59E-05 | 0.33 |
| 6 | 73323337 - 73687246 | CGAS | cis | -0.83 | 0.42 | 0.21 | 3.64E-05 | 0.33 |
| 3 | 54046214 - 54132558 | CACNA2D3 | cis | 0.57 | 1.77 | 0.14 | 4.16E-05 | 0.33 |
| 17 | 57953222 - 58107792 | SRSF1 | cis | -1.14 | 0.32 | 0.28 | 4.18E-05 | 0.33 |
| 6 | 165903009 - 166262103 | Unknown | - | -0.28 | 0.76 | 0.07 | 4.25E-05 | 0.33 |
| 6 | 168515512 - 168540991 | SMOC2 | cis | 1.57 | 4.79 | 0.38 | 4.52E-05 | 0.33 |
| 3 | 119384733 - 119555854 | TMEM39A | cis | -0.44 | 0.65 | 0.11 | 4.75E-05 | 0.33 |
| 12 | 128820252 - 129150901 | GLT1D1 | cis | 0.21 | 1.23 | 0.05 | 5.07E-05 | 0.34 |
| 1 | 10830875 - 10840291 | CASZ1 | cis | -1.96 | 0.14 | 0.50 | 9.29E-05 | 0.37 |

Associations are ordered by P value (from lowest to highest) and only those with $P<{1 x 10}^{-4}$ before FDR correction are presented. HR=Hazard Ratio, SE=standard error, FDR=False Discovery Rate.

## Supplementary Table 5 Associations between mQTLs and CRC-specific survival for participants diagnosed with CRC.

| **Chromosome** | **Region** | **Gene** | **mQTL type** | **Coefficient** | **HR** | **SE** | **P value** | **P, FDR** |
| --- | --- | --- | --- | --- | --- | --- | --- | --- |
| 16 | 57701610 - 57796223 | KATNB1 | cis | 0.95 | 2.60 | 0.19 | 7.11E-07 | 0.08 |
| 5 | 6566491 - 6762313 | LINC01018 | cis | -0.65 | 0.52 | 0.15 | 9.50E-06 | 0.24 |
| 1 | 155269833 - 156151299 | LMNA | cis | -1.32 | 0.27 | 0.30 | 9.83E-06 | 0.24 |
| 13 | 31126786 - 31333304 | HSPH1 | cis | -1.42 | 0.24 | 0.33 | 1.36E-05 | 0.24 |
| 7 | 38209042 - 39142602 | AMPH | cis | -0.51 | 0.60 | 0.12 | 1.45E-05 | 0.24 |
| 7 | 33002578 - 33514332 | BBS9 | cis | -0.78 | 0.46 | 0.18 | 1.52E-05 | 0.24 |
| 5 | 627928 - 627928 | Unknown | - | 1.79 | 5.99 | 0.42 | 1.75E-05 | 0.24 |
| 17 | 31007893 - 31009071 | RNF135 | cis | 3.03 | 20.60 | 0.70 | 1.76E-05 | 0.24 |
| 10 | 102155271 - 103717293 | NT5C2 | cis | 0.24 | 1.27 | 0.06 | 1.79E-05 | 0.24 |
| 6 | 40792477 - 41484103 | Unknown | - | -0.20 | 0.82 | 0.05 | 2.26E-05 | 0.24 |
| 7 | 33092117 - 33684939 | BBS9 | cis | -1.40 | 0.25 | 0.33 | 2.50E-05 | 0.24 |
| 7 | 128381575 - 128696139 | HILPDA-AS1 | cis | -1.12 | 0.33 | 0.27 | 2.66E-05 | 0.24 |
| 7 | 128379693 - 128776533 | IMPDH1 | cis | 0.65 | 1.92 | 0.16 | 2.71E-05 | 0.24 |
| 6 | 40961505 - 41412385 | Unknown | - | -0.59 | 0.56 | 0.14 | 2.98E-05 | 0.24 |
| 16 | 57701610 - 57738929 | KATNB1 | cis | -1.86 | 0.16 | 0.45 | 3.08E-05 | 0.24 |
| 17 | 8026459 - 8087771 | Unknown | cis | 1.06 | 2.87 | 0.26 | 3.66E-05 | 0.25 |
| 17 | 35445531 - 35451224 | SLFN11 | cis | 2.97 | 19.48 | 0.72 | 3.68E-05 | 0.25 |
| 11 | 116650470 - 117371044 | PAFAH1B2 | cis | -0.50 | 0.60 | 0.12 | 4.44E-05 | 0.25 |
| 7 | 100543824 - 102025057 | COL26A1 | cis | 0.27 | 1.31 | 0.07 | 4.49E-05 | 0.25 |
| 6 | 73323337 - 73687246 | CGAS | cis | -1.05 | 0.35 | 0.26 | 4.55E-05 | 0.25 |
| 13 | 31131442 - 31244747 | Unknown | - | 2.56 | 12.97 | 0.63 | 4.69E-05 | 0.25 |
| 2 | 9631071 - 9647596 | Unknown | - | 0.98 | 2.67 | 0.24 | 4.88E-05 | 0.25 |
| 19 | 53978080 - 54173662 | OSCAR | cis | 0.26 | 1.30 | 0.06 | 4.94E-05 | 0.25 |
| 5 | 2565240 - 2578507 | Unknown | - | 1.02 | 2.78 | 0.25 | 5.22E-05 | 0.25 |
| 17 | 46919257 - 48718189 | Unknown | - | -0.18 | 0.84 | 0.04 | 5.28E-05 | 0.25 |

Associations are ordered by P value (from lowest to highest) and only those with $P<{1 x 10}^{-4}$ before FDR correction are presented. HR=Hazard Ratio, SE=standard error, FDR=False Discovery Rate.

Supplementary Table 5 (continuation) Associations between mQTLs and CRC-specific survival for participants diagnosed with CRC.

| **Chromosome** | **Region** | **Gene** | **mQTL type** | **Coefficient** | **HR** | **SE** | **P value** | **P, FDR** |
| --- | --- | --- | --- | --- | --- | --- | --- | --- |
| 5 | 54967862 - 56081174 | MCIDAS | cis | 0.32 | 1.38 | 0.08 | 5.79E-05 | 0.26 |
| 16 | 86201922 - 86307154 | Unknown | - | 0.46 | 1.58 | 0.11 | 6.33E-05 | 0.28 |
| 16 | 88651549 - 90076051 | ACSF3 | cis | -0.24 | 0.79 | 0.06 | 6.57E-05 | 0.28 |
| 10 | 28099821 - 28359072 | MPP7 | cis | -1.35 | 0.26 | 0.34 | 7.23E-05 | 0.30 |
| 16 | 57695495 - 57727799 | DRC7 | cis | 1.20 | 3.32 | 0.31 | 8.71E-05 | 0.35 |
| 14 | 23080110 - 23168657 | SLC7A8 | cis | 0.44 | 1.56 | 0.11 | 8.99E-05 | 0.35 |
| 8 | 60280591 - 60316292 | CA8 | cis | 3.03 | 20.61 | 0.77 | 9.36E-05 | 0.35 |

Associations are ordered by P value (from lowest to highest) and only those with $P<{1 x 10}^{-4}$ before FDR correction are presented. HR=Hazard Ratio, SE=standard error, FDR=False Discovery Rate.

Supplementary Table 6 Associations between mQTLs and recurrence for participants diagnosed with CRC.

| **Chromosome** | **Region** | **Gene** | **mQTL type** | **Coefficient** | **HR** | **SE** | **P value** | **P, FDR** |
| --- | --- | --- | --- | --- | --- | --- | --- | --- |
| 2 | 222719463 - 222783572 | Unknown | - | 3.11 | 22.33 | 0.64 | 1.27E-06 | 0.10 |
| 14 | 73282200 - 74976400 | VSX2 | cis | 0.44 | 1.56 | 0.09 | 2.18E-06 | 0.10 |
| 3 | 128498656 - 128507913 | Unknown | - | 1.87 | 6.51 | 0.40 | 2.60E-06 | 0.10 |
| 5 | 174430648 - 174581416 | Unknown | - | 1.41 | 4.09 | 0.31 | 5.83E-06 | 0.14 |
| 10 | 48294959 - 48481483 | MAPK8 | cis | -2.01 | 0.13 | 0.44 | 5.86E-06 | 0.14 |
| 11 | 128947340 - 129303695 | ARHGAP32 | cis | 3.15 | 23.36 | 0.71 | 8.23E-06 | 0.16 |
| 2 | 216054364 - 216054364 | TMEM169 | cis | 2.93 | 18.72 | 0.67 | 1.09E-05 | 0.16 |
| 17 | 12048678 - 12093046 | MAP2K4 | cis | -2.26 | 0.10 | 0.52 | 1.14E-05 | 0.16 |
| 14 | 74244383 - 74338611 | ABCD4 | cis | -1.53 | 0.22 | 0.35 | 1.22E-05 | 0.16 |
| 1 | 113677683 - 113938630 | HIPK1-AS1 | cis | 3.58 | 35.98 | 0.83 | 1.75E-05 | 0.21 |
| 16 | 46967714 - 48106421 | DUSP22 | trans | -1.36 | 0.26 | 0.32 | 2.20E-05 | 0.24 |
| 1 | 54796778 - 55583129 | PCSK9 | cis | 0.27 | 1.31 | 0.06 | 2.51E-05 | 0.25 |
| 14 | 73668273 - 74498927 | ABCD4 | cis | -0.55 | 0.58 | 0.13 | 4.05E-05 | 0.37 |
| 3 | 14572428 -14572428 | Unknown | - | -3.02 | 0.05 | 0.74 | 4.49E-05 | 0.38 |
| 3 | 14495861 - 14567355 | GRIP2 | cis | -1.37 | 0.25 | 0.34 | 5.02E-05 | 0.38 |
| 11 | 128625170 - 129073661 | Unknown | - | 0.67 | 1.95 | 0.16 | 5.21E-05 | 0.38 |
| 17 | 11927561 - 12107363 | MAP2K4 | cis | -1.50 | 0.22 | 0.37 | 5.54E-05 | 0.38 |
| 14 | 74208193 - 74319835 | VSX2 | cis | 1.62 | 5.03 | 0.40 | 5.96E-05 | 0.38 |
| 11 | 128625170 - 129073661 | Unknown | - | 0.50 | 1.64 | 0.12 | 6.34E-05 | 0.38 |
| 3 | 13787960 - 14616091 | GRIP2 | cis | -0.42 | 0.66 | 0.10 | 6.45E-05 | 0.38 |
| 8 | 59051969 - 59651191 | TOX-DT | cis | 0.92 | 2.50 | 0.23 | 8.13E-05 | 0.41 |
| 16 | 48891741 - 48989938 | Unknown | - | 0.54 | 1.72 | 0.14 | 8.15E-05 | 0.41 |
| 20 | 50644662 - 50936367 | Unknown | - | 0.68 | 1.97 | 0.17 | 8.42E-05 | 0.41 |
| 10 | 30817750 - 31164963 | ZNF438 | cis | 0.76 | 2.13 | 0.19 | 8.46E-05 | 0.41 |
| 22 | 37215352 - 37215472 | SSTR3 | cis | -2.84 | 0.06 | 0.72 | 8.63E-05 | 0.41 |
| 2 | 231436075 - 231561242 | NCL | cis | 1.24 | 3.46 | 0.32 | 9.73E-05 | 0.43 |
| 5 | 66928192 - 66979224 | MAST4 | cis | 1.25 | 3.49 | 0.32 | 9.85E-05 | 0.43 |

Associations are ordered by P value (from lowest to highest) and only those with $P<{1 x 10}^{-4}$ before FDR correction are presented. HR=Hazard Ratio, SE=standard error, FDR=False Discovery Rate.

Supplementary Table 7 Colocalisation of mQTLs and CRC meta-GWAS data for candidate region in chromosome 4 mapped to PPA2 gene (boundaries for region:104940737- 105558268)

| **Probe** | **No. SNPs in common with meta-GWAS** | **PP H0** | **PP H1** | **PP H2** | **PP H3** | **PP H4** | **Top colocalised SNP(s)** |
| --- | --- | --- | --- | --- | --- | --- | --- |
| cg21545890 | 22 | <0.01 | 0.39 | <0.01 | 0.10 | 0.51 |  |
| cg09289469 | 318 | <0.01 | <0.01 | <0.01 | 1.00 | <0.01 |  |
| cg20073050 | 160 | <0.01 | <0.01 | <0.01 | 1.00 | <0.01 |  |
| cg23745290 | 148 | <0.01 | 0.99 | <0.01 | <0.01 | 0.01 |  |
| cg12147105 | 194 | <0.01 | 0.99 | <0.01 | <0.01 | 0.01 |  |

PP=posterior probability for each one of the hypothesis tested calculated with colco.abf() function. Shaded area presents the highest PP for that region. Probe in red is presented in the main text. Probes are presented ordered by p value for the association with CRC risk (from lowest to highest). Top colocalised SNP(s) only presented when PPH4>0.80.

Supplementary Table 8 Colocalisation of mQTLs and CRC meta-GWAS data for candidate region in chromosome 8 mapped to POU5F1B, CASC8 and PCAT1 genes (boundaries for region: 127383349- 127454743)

| **Probe** | **No. SNPs in common with meta-GWAS** | **PP H0** | **PP H1** | **PP H2** | **PP H3** | **PP H4** | **Top colocalised SNP(s)** |
| --- | --- | --- | --- | --- | --- | --- | --- |
| cg14289643 | 92 | <0.01 | <0.01 | <0.01 | 1.00 | <0.01 |  |
| cg25305703 | 23 | <0.01 | <0.01 | <0.01 | 1 | <0.01 |  |
| cg14437045 | 23 | <0.01 | <0.01 | <0.01 | 1 | <0.01 |  |

PP=posterior probability for each one of the hypothesis tested calculated with colco.abf() function. Shaded area presents the highest PP for that region. Probe in red is presented in the main text. Probes are presented ordered by p value for the association with CRC risk (from lowest to highest). Top colocalised SNP(s) only presented when PPH4>0.80.

Supplementary Table 9 Colocalisation of mQTLs and CRC meta-GWAS data for candidate region in chromosome 11 mapped to POU2AF2 (C11orf53), COLCA1 and POU2AF3 (COLCA2) genes (boundaries for region: 111120465- 111805145)

| **Probe** | **No. SNPs in common with meta-GWAS** | **PP H0** | **PP H1** | **PP H2** | **PP H3** | **PP H4** | **Top colocalised SNP(s)** |
| --- | --- | --- | --- | --- | --- | --- | --- |
| cg25129781 | 111 | <0.01 | <0.01 | <0.01 | 0.18 | 0.82 | rs3087967 |
| cg09213929 | 74 | <0.01 | <0.01 | <0.01 | 0.32 | 0.68 | rs4608113 |
| cg26459819 | 201 | <0.01 | <0.01 | <0.01 | <0.01 | 1.00 | rs3087967 |
| cg10045354 | 186 | <0.01 | <0.01 | <0.01 | 0.08 | 0.92 | rs7130173 |
| cg21645554 | 193 | <0.01 | <0.01 | <0.01 | <0.01 | 0.99 | rs3087967 |
| cg06978117 | 47 | <0.01 | <0.01 | <0.01 | 0.03 | 0.97 | rs10789819 |

PP=posterior probability for each one of the hypothesis tested calculated with colco.abf() function. Shaded area presents the highest PP for that region. Probes in red are presented in the main text. Probes are presented ordered by p value for the association with CRC risk (from lowest to highest). Top colocalised SNP(s) only presented when PPH4>0.80.

Supplementary Table 10 Colocalisation of mQTLs and CRC meta-GWAS data for candidate region in chromosome 12 mapped to POU6F1 gene (boundaries for region: 50665404- 51372735)

| **Probe** | **No. SNPs in common with meta-GWAS** | **PP H0** | **PP H1** | **PP H2** | **PP H3** | **PP H4** | **Top colocalised SNP(s)** |
| --- | --- | --- | --- | --- | --- | --- | --- |
| cg22163059 | 163 | <0.01 | <0.01 | <0.01 | 1.00 | <0.01 |  |
| cg18912120 | 10 | <0.01 | 1 | <0.01 | <0.01 | <0.01 |  |
| cg13217116 | 32 | <0.01 | 0.99 | <0.01 | <0.01 | <0.01 |  |
| cg23530981 | 15 | <0.01 | 0.99 | <0.01 | <0.01 | 0.01 |  |
| cg12163867 | 579 | <0.01 | <0.01 | <0.01 | 0.08 | 0.92 | rs57602501 |

PP=posterior probability for each one of the hypothesis tested calculated with colco.abf() function. Shaded area presents the highest PP for that region. Probe in red is presented in the main text. Probes are presented ordered by p value for the association with CRC risk (from lowest to highest). Top colocalised SNP(s) only presented when PPH4>0.80.

Supplementary Table 11 Colocalisation of mQTLs and CRC meta-GWAS data for candidate region in chromosome 14 mapped to MDGA2 gene (boundaries for region: 47180232- 47709403)

| **Probe** | **No. SNPs in common with meta-GWAS** | **PP H0** | **PP H1** | **PP H2** | **PP H3** | **PP H4** | **Top colocalised SNP(s)** |
| --- | --- | --- | --- | --- | --- | --- | --- |
| cg22964621 | 586 | <0.01 | 0.99 | <0.01 | <0.01 | <0.01 |  |
| cg01098491 | 179 | <0.01 | 0.98 | <0.01 | <0.01 | 0.02 |  |
| cg23629393 | 465 | <0.01 | 0.99 | <0.01 | <0.01 | <0.01 |  |
| cg26010412 | 247 | <0.01 | 0.98 | <0.01 | <0.01 | 0.01 |  |
| cg02126950 | 92 | <0.01 | 0.98 | <0.01 | <0.01 | 0.02 |  |
| cg13687915 | 174 | <0.01 | 1 | <0.01 | <0.01 | <0.01 |  |
| cg05278500 | 12 | <0.01 | 0.98 | <0.01 | <0.01 | 0.02 |  |
| cg08217024 | 480 | <0.01 | 0.99 | <0.01 | <0.01 | <0.01 |  |

PP=posterior probability for each one of the hypothesis tested calculated with colco.abf() function. Shaded area presents the highest PP for that region. Probe in red is presented in the main text. Probes are presented ordered by p value for the association with CRC risk (from lowest to highest). Top colocalised SNP(s) only presented when PPH4>0.80.

Supplementary Table 12 Colocalisation of mQTLs and CRC meta-GWAS data for candidate region in chromosome 15 mapped to GREM1 gene (boundaries for region: 47180232- 47709403)

| **Probe** | **No. SNPs in common with meta-GWAS** | **PP H0** | **PP H1** | **PP H2** | **PP H3** | **PP H4** | **Top colocalised SNP(s)** |
| --- | --- | --- | --- | --- | --- | --- | --- |
| cg21924449 | 102 | <0.01 | <0.01 | <0.01 | 1.00 | <0.01 |  |
| cg14605150 | 21 | <0.01 | <0.01 | <0.01 | 1 | <0.01 |  |
| cg07179000 | 413 | <0.01 | <0.01 | <0.01 | 0.99 | 0.01 |  |
| cg09392615 | 384 | <0.01 | <0.01 | <0.01 | 0.31 | 0.69 |  |
| cg18145505 | 117 | <0.01 | <0.01 | <0.01 | 0.06 | 0.94 | rs2293582 |
| cg03884132 | 47 | <0.01 | <0.01 | <0.01 | <0.01 | 1 | rs1919364 |
| cg09656020 | 27 | <0.01 | <0.01 | <0.01 | 1 | <0.01 |  |

PP=posterior probability for each one of the hypothesis tested calculated with colco.abf() function. Shaded area presents the highest PP for that region. Probe in red is presented in the main text. Probes are presented ordered by p value for the association with CRC risk (from lowest to highest). Top colocalised SNP(s) only presented when PPH4>0.80.

Supplementary Table 13 Colocalisation of mQTLs and CRC meta-GWAS data for candidate region in chromosome 17 mapped to STARD3 gene (boundaries for region38742575- 40487962)

| **Probe** | **No. SNPs in common with meta-GWAS** | **PP H0** | **PP H1** | **PP H2** | **PP H3** | **PP H4** | **Top colocalised SNP(s)** |
| --- | --- | --- | --- | --- | --- | --- | --- |
| cg00129232 | 1606 | <0.01 | <0.01 | <0.01 | 0.99 | <0.01 |  |
| cg14187895 | 1191 | <0.01 | <0.01 | <0.01 | 0.15 | 0.85 | rs2313171 |
| cg03815147 | 1644 | <0.01 | <0.01 | <0.01 | 0.88 | 0.12 |  |
| cg19758448 | 1106 | <0.01 | <0.01 | <0.01 | 0.65 | 0.35 |  |
| cg26615017 | 585 | <0.01 | <0.01 | <0.01 | 0.29 | 0.77 |  |
| cg16557858 | 1103 | <0.01 | <0.01 | <0.01 | 0.12 | 0.88 | rs903501 |
| cg04429812 | 851 | <0.01 | <0.01 | <0.01 | 0.28 | 0.72 |  |
| cg05512684 | 479 | <0.01 | <0.01 | <0.01 | 0.52 | 0.48 |  |
| cg19586165 | 580 | <0.01 | <0.01 | <0.01 | 0.32 | 0.68 |  |
| cg22243000 | 127 | <0.01 | 0.01 | <0.01 | 0.99 | <0.01 |  |
| cg03572260 | 72 | <0.01 | <0.01 | <0.01 | 0.29 | 0.70 |  |
| cg16027267 | 189 | <0.01 | 0.56 | <0.01 | 0.14 | 0.29 |  |
| cg02082887 | 72 | <0.01 | 0.96 | <0.01 | 0.03 | 0.01 |  |
| cg10986043 | 440 | <0.01 | <0.01 | <0.01 | 0.20 | 0.80 |  |
| cg01799856 | 1173 | <0.01 | <0.01 | <0.01 | 0.97 | 0.03 |  |
| cg24526433 | 217 | <0.01 | 0.09 | <0.01 | 0.65 | 0.26 |  |
| cg06247837 | 383 | <0.01 | <0.01 | <0.01 | 0.20 | 0.79 |  |

PP=posterior probability for each one of the hypothesis tested calculated with colco.abf() function. Shaded area presents the highest PP for that region. Probe in red is presented in the main text. Probes are presented ordered by p value for the association with CRC risk (from lowest to highest). Top colocalised SNP(s) only presented when PPH4>0.80.

Supplementary Table 14 Colocalisation of mQTLs and CRC meta-GWAS data for candidate region in chromosome 18 mapped to CTIF gene (boundaries for region: 48839047- 48934015)

| **Probe** | **No. SNPs in common with meta-GWAS** | **PP H0** | **PP H1** | **PP H2** | **PP H3** | **PP H4** | **Top colocalised SNP(s)** |
| --- | --- | --- | --- | --- | --- | --- | --- |
| cg13840032 | 10 | <0.01 | <0.01 | <0.01 | 1 | <0.01 |  |
| cg19811144 | 23 | <0.01 | 1 | <0.01 | <0.01 | <0.01 |  |
| cg12419932 | 35 | <0.01 | 1 | <0.01 | <0.01 | <0.01 |  |
| cg21602842 | 63 | <0.01 | 1 | <0.01 | <0.01 | <0.01 |  |

PP=posterior probability for each one of the hypothesis tested calculated with colco.abf() function. Shaded area presents the highest PP for that region. Probe in red is presented in the main text. Probes are presented ordered by p value for the association with CRC risk (from lowest to highest). Top colocalised SNP(s) only presented when PPH4>0.80.

Supplementary Table 15 Colocalisation of mQTLs and CRC meta-GWAS data for candidate region in chromosome 20 mapped to CABLES2 and LAMA5 genes (boundaries for region: 61913515- 62472409)

| **Probe** | **No. SNPs in common with meta-GWAS** | **PP H0** | **PP H1** | **PP H2** | **PP H3** | **PP H4** | **Top colocalised SNP(s)** |
| --- | --- | --- | --- | --- | --- | --- | --- |
| cg22601191 | 537 | <0.01 | <0.01 | <0.01 | 0.05 | 0.94 | rs1741640 |
| cg15193198 | 127 | <0.01 | <0.01 | <0.01 | 1 | <0.01 |  |
| cg06026331 | 529 | <0.01 | <0.01 | <0.01 | 1 | <0.01 |  |
| cg22307297 | 471 | <0.01 | <0.01 | <0.01 | 0.06 | 0.94 | rs1741640 |
| cg01379171 | 17 | <0.01 | <0.01 | <0.01 | 0.02 | 0.98 | rs2427313 |
| cg10038901 | 274 | <0.01 | <0.01 | <0.01 | 1 | <0.01 |  |
| cg04517374 | 162 | <0.01 | <0.01 | <0.01 | 1 | <0.01 |  |
| cg02605258 | 283 | <0.01 | <0.01 | <0.01 | 1 | <0.01 |  |
| cg18668449 | 142 | <0.01 | <0.01 | <0.01 | 1 | <0.01 |  |
| cg04969764 | 122 | <0.01 | <0.01 | <0.01 | 0.18 | 0.82 | rs1741640 |
| cg06197360 | 425 | <0.01 | <0.01 | <0.01 | 1 | <0.01 |  |

PP=posterior probability for each one of the hypothesis tested calculated with colco.abf() function. Shaded area presents the highest PP for that region. Probe in red is presented in the main text. Probes are presented ordered by p value for the association with CRC risk (from lowest to highest). Top colocalised SNP(s) only presented when PPH4>0.80.

Supplementary Table 16 Comparison of results between this study (looking at the association of mQTLs with CRC risk) and CRC meta-GWAS.

| **Chr** | **Region*** | **Gene (s)** | **SNPs in meta-GWAS within that region** | **Position (GRCh38)** | **Closer effector gene identified in meta-GWAS for that region** | **Gene start position** | **SNP found to colocalise between both studies** | **LD with SNP from meta-GWAS for European population** |
| --- | --- | --- | --- | --- | --- | --- | --- | --- |
| 4 | 104940737- 105558268 | PPA2 | rs2007403 | 105210053 | TET2 | 105145875 |  |  |
| 6 | 36402477-36758526 | PANDAR and LAP3P2 | rs9470361 | 36655602 | *CDKN1A* | 36676463 | rs9470361 |  |
| 8 | 127383349- 127454743 | POU5F1B, CASC8 and PCAT1 | rs6983267 | 127401060 | *POU5F1B* | 127413447 |  |  |
|  |  |  | rs7013278 | 127402647 |  |  |  |  |
| 11 | 111120465- 111805145 | *POU2AF2 (*C11orf53), COLCA1,  *POU2AF3 (*COLCA2) | rs3087967 | 111286111 | *C11orf53 and COLCA2* | 111245725 | rs3087967 |  |
| 12 | 50665404- 51372735 | POU6F1 | rs11169572 | 50823107 | *LIMA1* | 50175788 | rs57602501 | R2:0.323; D':1 |
| 14 | 47180232- 47709403 | MDGA2 |  |  | No gene within 5MB |  |  |  |
| 15 | 32694234- 32814035 | GREM1 | rs1554865 | 32707605 | *GREM1* | 32718004 | rs2293581 | R2:0.629; D':0.82 |
|  |  |  | rs1919364 | 32717373 |  |  |  |  |
| 17 | 38742575- 40487962 | STARD3 |  |  | No gene within 20MB |  | rs2313171 |  |
| 18 | 48839047- 48934015 | CTIF | rs2337113 | 48925957 | *ACAA2* | 49782164 |  |  |
| 20 | 61913515- 62472409 | CABLES2, LAMA5 | rs1741640 | 62357358 | *CABLES2* | 62388634 | rs1741640 |  |

Genes in green have been found as effector genes in meta-GWAS. Genes in yellow have been found as effector genes within that same region but a different gene was identified in our study. For regions in red, no gene (or single SNP) has been listed in meta-GWAS.

Supplementary Figure 2 Locus plots for probe cg02730678 and meta-GWAS data within LAP3P2/PANDAR genes region that were found to colocalised. A diagram of the LD pattern is also presented. Top colocalised SNP is rs9470361.


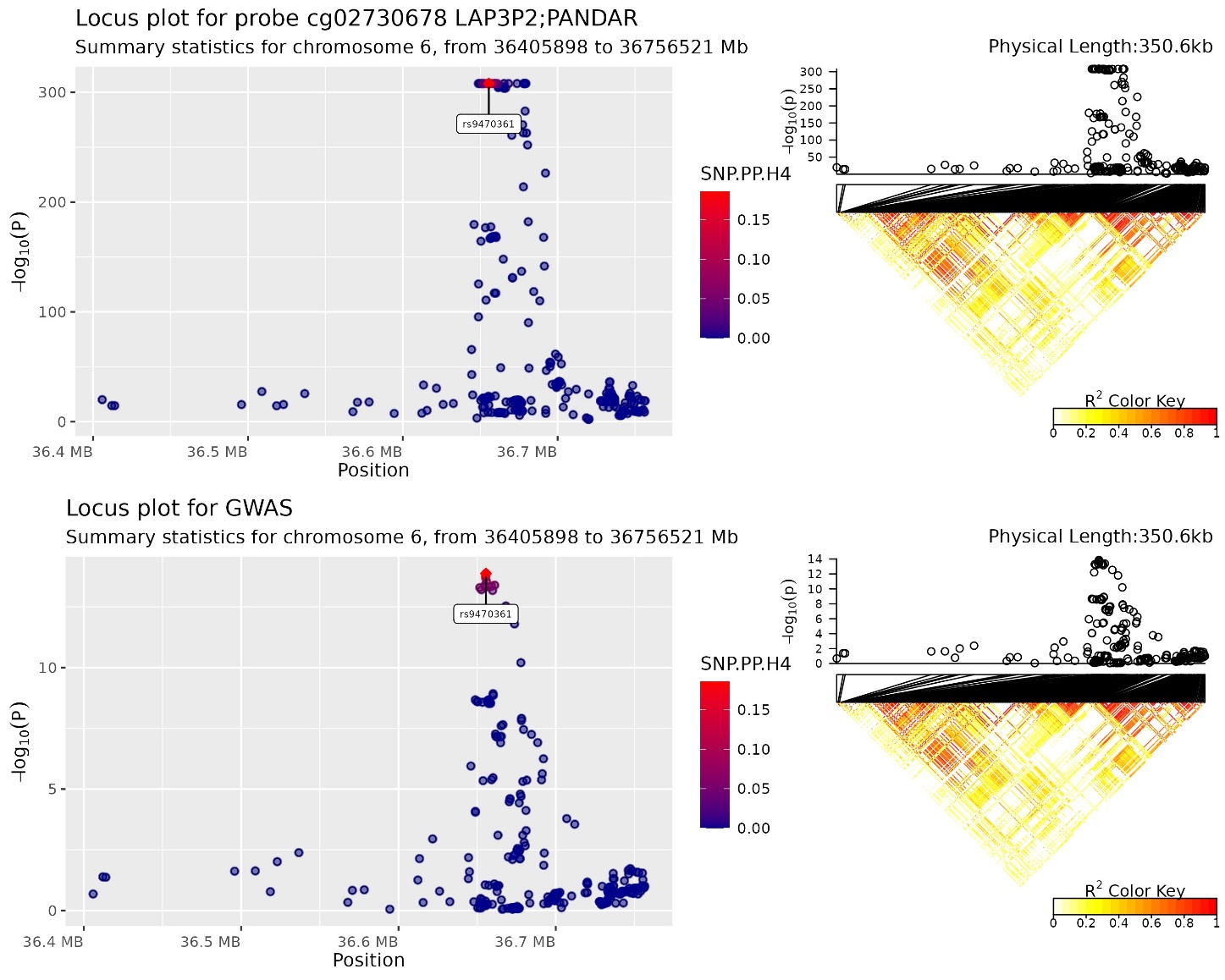


Supplementary Figure 3 Locus plots for probe cg12163867and meta-GWAS data within POU6F1 gene region that were found to colocalised. A diagram of the LD pattern is also presented. Top colocalised SNP is rs57602501.


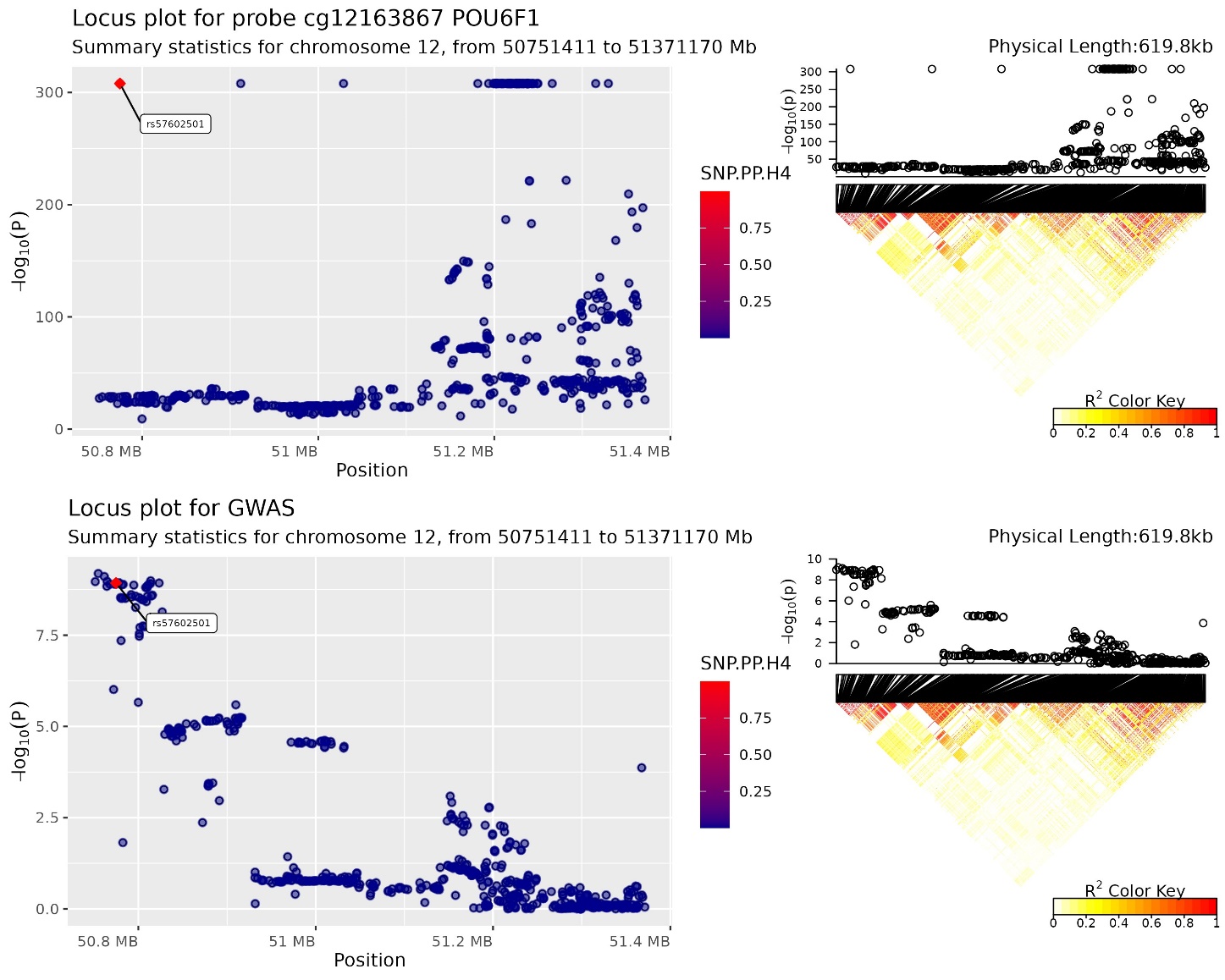


Supplementary Figure 4 Locus plots for probe cg18145505and meta-GWAS data within GREM1 gene region that were found to colocalised. A diagram of the LD pattern is also presented. Top colocalised SNP is rs2293581.


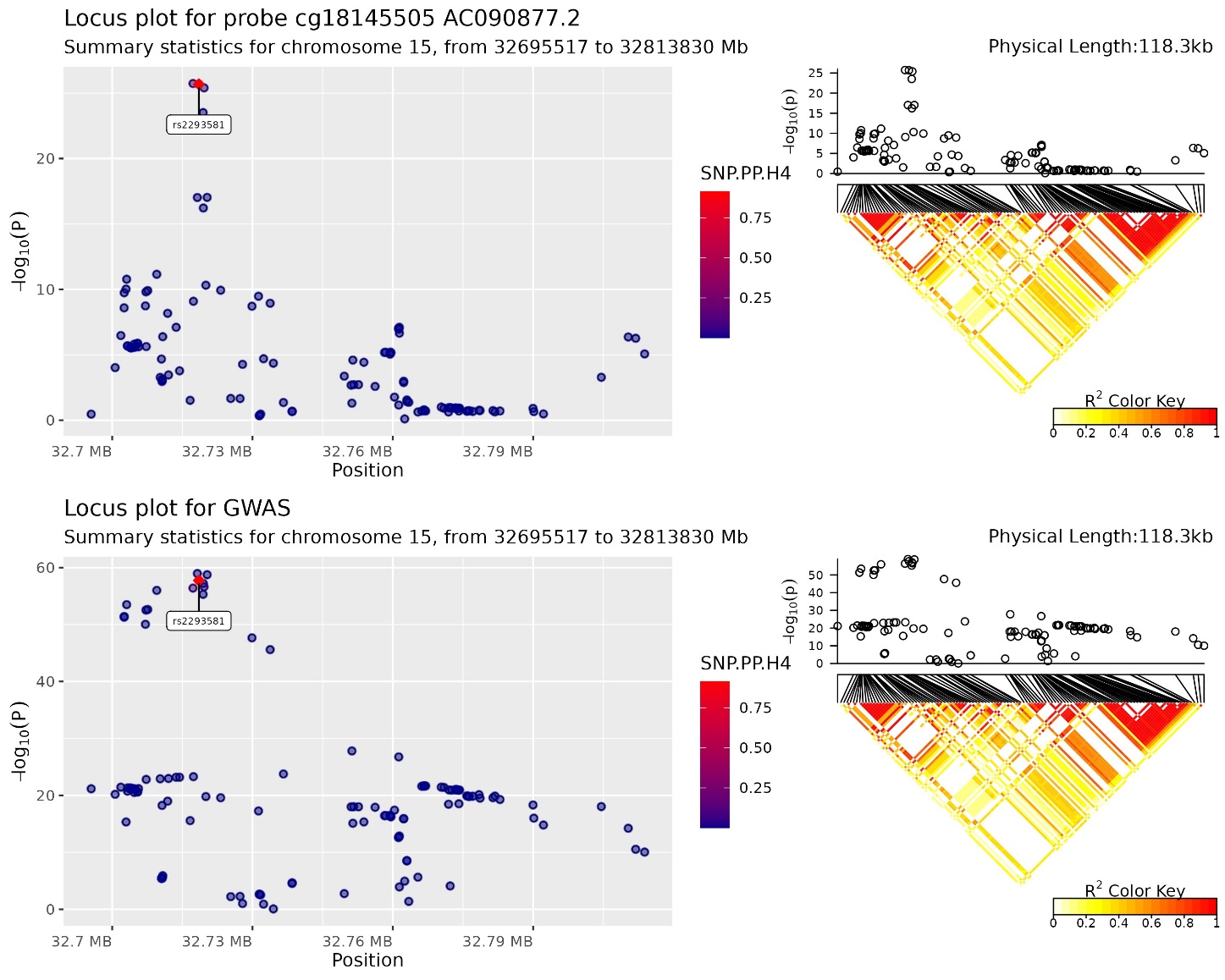


Supplementary Figure 5 Locus plots for probe cg14187895and meta-GWAS data within STARD3 gene region that were found to colocalised. A diagram of the LD pattern is also presented. Top colocalised SNP is rs2313171.


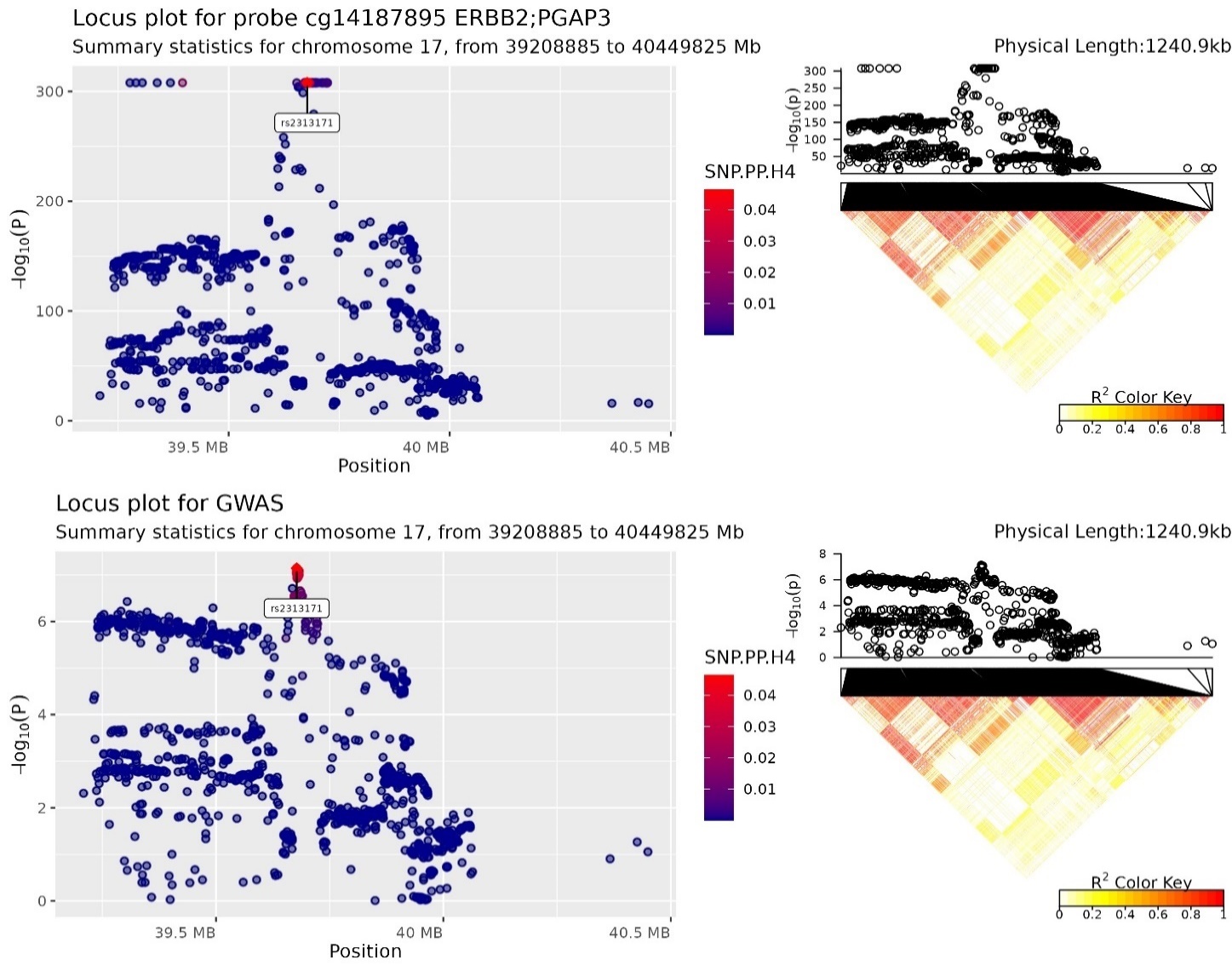


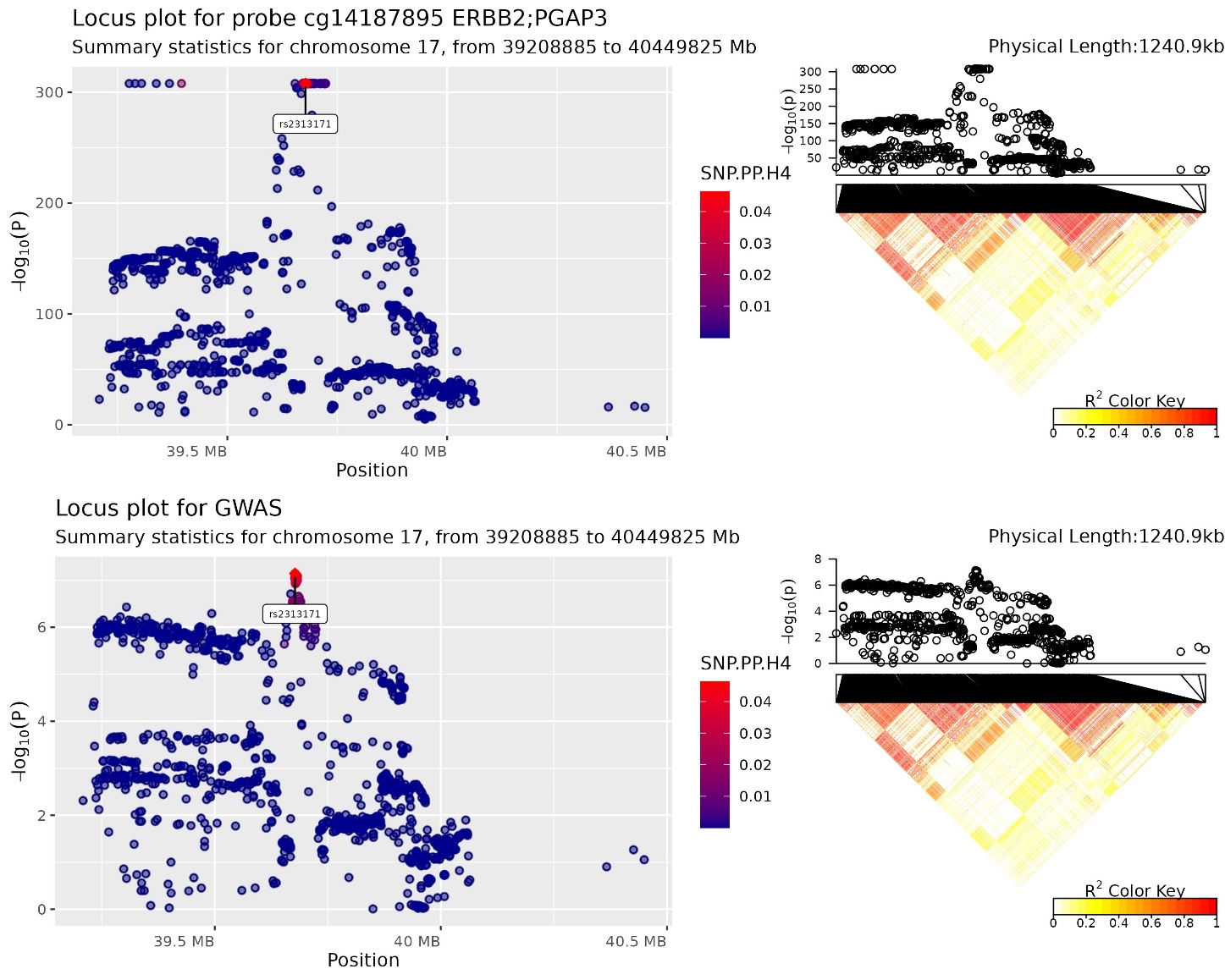


Supplementary Figure 6 Locus plots for probe cg22601191and meta-GWAS data within CABLES2/LAMA5 genes region that were found to colocalised. A diagram of the LD pattern is also presented. Top colocalised SNP is rs1741640.


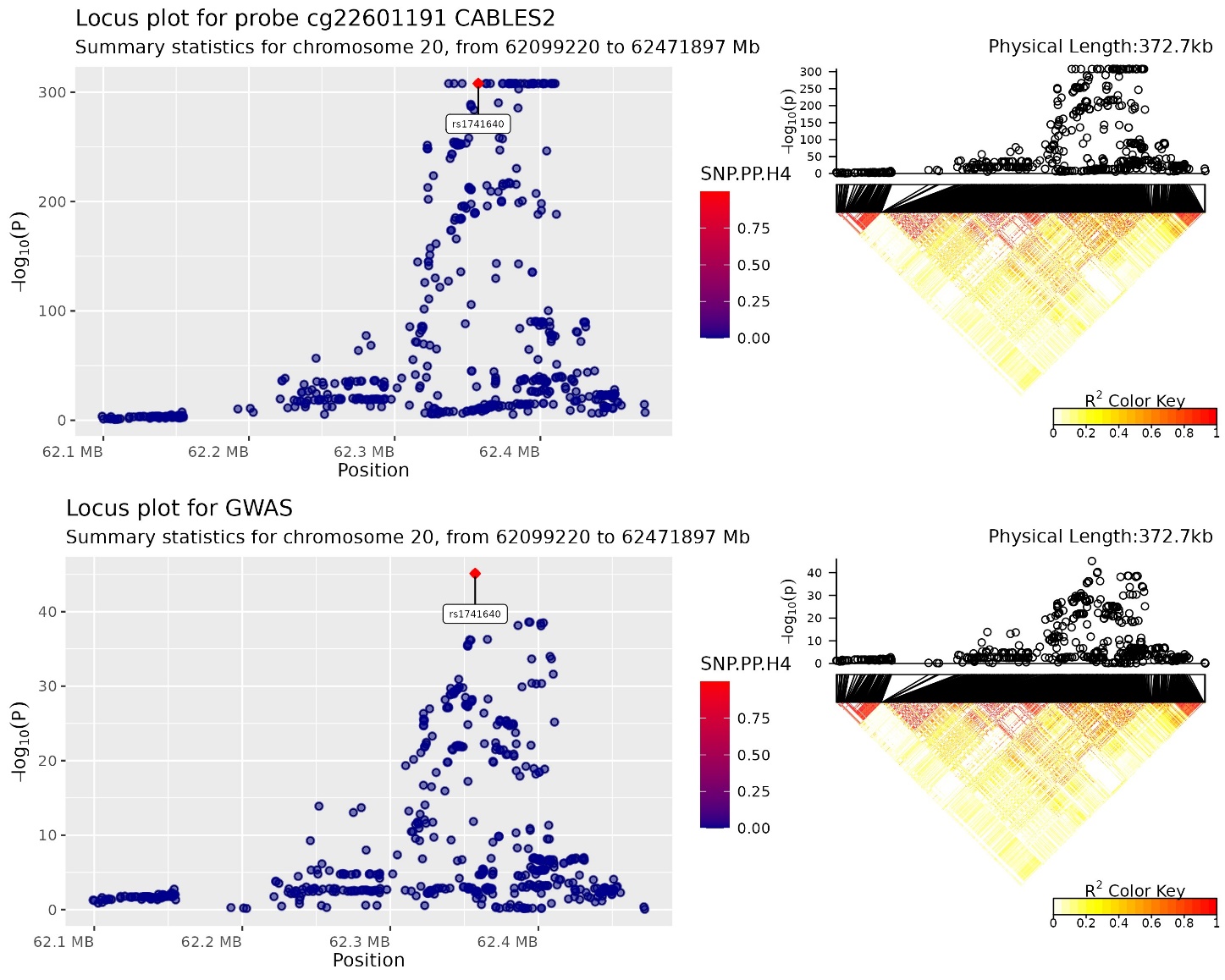


References

1. Li X, Timofeeva M, Spiliopoulou A, McKeigue P, He Y, Zhang X, et al. Prediction of colorectal cancer risk based on profiling with common genetic variants. International journal of cancer. 2020;147(12):3431-7.
